# The RNA interference pathway affects midgut infection- and escape barriers for Sindbis virus in *Aedes aegypti*

**DOI:** 10.1186/1471-2180-10-130

**Published:** 2010-04-28

**Authors:** Cynthia CH Khoo, Joseph Piper, Irma Sanchez-Vargas, Ken E Olson, Alexander WE Franz

**Affiliations:** 1 Arthropod-Borne and Infectious Diseases Laboratory, Department of Microbiology, Immunology and Pathology, Colorado State University, Fort Collins, CO 80523 USA

## Abstract

**Background:**

The RNA interference (RNAi) pathway acts as an innate antiviral immune response in *Aedes aegypti*, modulating arbovirus infection of mosquitoes. Sindbis virus (SINV; family: *Togaviridae*, genus: *Alphavirus*) is an arbovirus that infects *Ae. aegypti *in the laboratory. SINV strain TR339 encounters a midgut escape barrier (MEB) during infection of *Ae. aegypti*. The nature of this barrier is not well understood. To investigate the role of the midgut as the central organ determining vector competence for arboviruses, we generated transgenic mosquitoes in which the RNAi pathway was impaired in midgut tissue of bloodfed females. We used these mosquitoes to reveal effects of RNAi impairment in the midgut on SINV replication, midgut infection and dissemination efficiencies, and mosquito longevity.

**Results:**

As a novel tool for studying arbovirus-mosquito interactions, we engineered a transgenic mosquito line with an impaired RNAi pathway in the midgut of bloodfed females by silencing expression of the *Aa*-*dcr2 *gene. In midgut tissue of the transgenic Carb/dcr16 line, *Aa*-*dcr2 *expression was reduced ~50% between 1-7 days post-bloodmeal (pbm) when compared to the recipient mosquito strain. After infection with SINV-TR339EGFP, *Aa*-*dcr2 *expression levels were enhanced in both mosquito strains. In the RNAi pathway impaired mosquito strain SINV titers and midgut infection rates were significantly higher at 7 days pbm. There was also a strong tendency for increased virus dissemination rates among the transgenic mosquitoes. Between 7-14 days pbm, SINV was diminished in midgut tissue of the transgenic mosquitoes. Transgenic impairment of the RNAi pathway and/or SINV infection did not affect longevity of the mosquitoes.

**Conclusions:**

We showed that RNAi impaired transgenic mosquitoes are a useful tool for studying arbovirus-mosquito interactions at the molecular level. Following ingestion by *Ae. aegypti*, the recombinant SINV-TR339EGFP was confronted with both MEB and a midgut infection barrier (MIB). Impairment of the RNAi pathway in the midgut strongly reduced both midgut barriers for the virus. This confirms that the endogenous RNAi pathway of *Ae. aegypti *modulates vector competence for SINV in the midgut. The RNAi pathway acts as a gatekeeper to the incoming virus by affecting infection rate of the midgut, intensity of infection, and dissemination from the midgut to secondary tissues.

## Background

The RNA interference (RNAi) pathway is an innate immune pathway of invertebrates such as nematodes, trypanosomes, hydra, planaria, and insects [1]. In mosquitoes, the RNAi pathway has been shown to act as an antiviral immune pathway that is able to effectively modulate the replication pattern of arthropod-borne viruses (arboviruses) [2-6]. It has been postulated that RNAi functions as a gatekeeper in mosquitoes, modulating arbovirus replication to allow virus transmission but preventing virus concentrations that could lead to fitness costs and pathogenic effects [6]. Consequently, RNAi is potentially a major factor determining the vector competence of mosquitoes for arboviruses.

Sindbis virus (SINV; family: *Togaviridae*; genus: *Alphavirus*) is an arbovirus with a positive sense single-stranded RNA genome. A dsRNA intermediate is formed during replication, which triggers the RNAi pathway causing homology-dependent destruction of viral RNA [3].  Since SINV is able to establish persistent infections in the mosquito, the virus must have developed strategies to cope with the antiviral RNAi pathway in the insect host. Potential RNAi evasion strategies for alphaviruses are active suppression of the RNAi pathway and - similar to flaviviruses - sequestration of the dsRNA replicative intermediate within cellular membrane structures [7]. Under natural conditions, SINV circulates between *Culex *sp. and birds with humans acting as dead end hosts [8]. However, in the laboratory the virus is transmissible by the well characterized mosquito vector *Aedes aegypti*, prompting researchers to use the SINV-*Ae. aegypti *combination as a model to study arbovirus-mosquito interactions at the molecular level. After ingestion of a viremic bloodmeal by a competent mosquito, SINV enters midgut epithelial cells and begins replicating [9]. From the midgut the virus disseminates to secondary tissues such as muscles surrounding the alimentary tract, fat body, hemocytes, nerve tissue, and finally salivary glands. Once SINV enters the saliva, the virus has completed its extrinsic incubation period and the mosquito is able to transmit the virus to a new host [9]. The TR339 strain of SINV is based on a consensus sequence derived from the type strain AR339 that has been isolated in Egypt [10-12]. For this study, we used a full-length infectious cDNA clone of the virus with the enhanced green fluorescent protein (EGFP) marker gene inserted downstream of a second subgenomic promoter [3]. After ingestion by females of the *Ae. aegypti *RexD strain, SINV-TR339 has been shown to encounter an escape barrier in the midgut (MEB); whereas reported midgut infection rates were >90%, dissemination rates only reached 40% [9,13].

Midgut infection barrier (MIB) and/or MEB have been observed for a number of other alphaviruses and for flaviviruses [14,15]. MIB prevents ingested arboviruses from entering and replicating in mesenteronal (midgut) cells, whereas MEB prevents virions from escaping from the basal lamina of midgut cells and disseminating to other tissues in the hemocoel. Often these barriers depend on the amount of virus ingested by the mosquito because the virus has to reach a certain threshold to either establish an infection in the midgut or to disseminate to other tissues [9,14-16]. Furthermore, dose-independent MIB or MEB have been reported, implying an incompatibility between arbovirus and vector at the midgut level, thus preventing arboviruses from entering or exiting the epithelial cells [13,17-20]. Until now, the molecular nature of MIB and MEB, which appears to depend on specific virus-mosquito strain combinations, is not well understood. However, recent correlation analysis of RNAi pathway genes with MIB and MEB combined with linkage mapping of *Aa-dcr2*, *Aa-r2d2*, and *Aa-ago2 *genes in the genome of *Ae. aegypti *suggests that MIB and MEB for dengue virus could be RNAi associated phenomena [21].

To investigate the nature of MIB and MEB for SINV-TR339EGFP in *Ae. aegypti*, we impaired the RNAi pathway in the mosquito midgut at a time point when the ingested virus is replicating in cells of the midgut epithelium. We expected that impairment of the RNAi pathway in the midgut of *Ae. aegypti *would allow the virus to overcome potential MIB and/or MEB and to increase its overall titer in the insect. We chose a transgenic approach to impair the RNAi pathway in the midgut of *Ae. aegypti *by generating mosquitoes expressing an inverted-repeat (IR) RNA derived from the RNAi pathway gene *Aa-dcr2 *under control of the bloodmeal inducible, midgut-specific *Ae. aegypti carboxypeptidase A *(*AeCPA*) promoter [22-25]. According to our strategy the midgut-specific IR effector would produce dsRNA in bloodfed females, triggering RNAi against *Aa-dcr2 *and eventually causing depletion of dicer2 protein in the midgut. This would cause impairment of the RNAi pathway in this tissue. Previously, it has been demonstrated that homology-dependent silencing of *dcr2 *can impair the RNAi mechanism in insect cells and in *Ae. aegypti *[26-28].

The objectives of this study are to generate transgenic *Ae. aegypti *mosquitoes with an impaired RNAi pathway in midgut tissue after ingestion of a bloodmeal, to assess vector competence of the transgenic mosquitoes for SINV-TR339EGFP with respect to possible effects on MIB and MEB, and to evaluate if midgut-specific impairment of the RNAi pathway reduces the survival rate of SINV-infected mosquitoes.

## Results

### Generation of transgenic *Ae. aegypti *expressing an IR RNA targeting *Aa-dcr2 *mRNA

**Figure 1:**
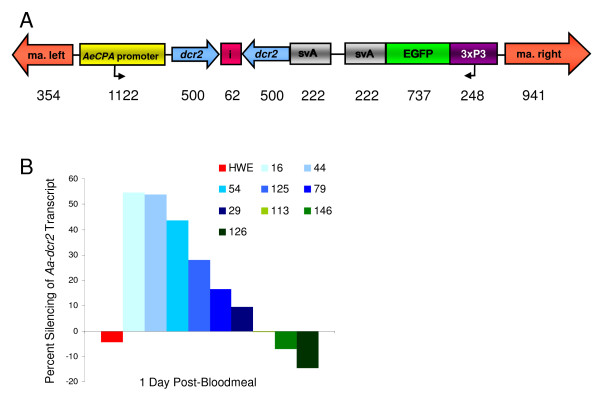
**Transgene design to silence *Aa-dcr2 *in the midgut of bloodfed females and molecular characterization of transgenic mosquito lines**. A) Five hundred base-pair (bp) cDNAs in sense and anti-sense orientations corresponding to a portion of *Aa-dcr2 *were used for the inverted repeat (IR) construction. Sense and anti-sense cDNA fragments of *Aa-dcr2 *were separated by the small intron of the *Aa-sialonkinin I *gene and placed downstream of the *Aa-carboxypeptidase A *promoter. A transcription termination signal derived from SV40 was added downstream of the IR construct. Numbers below the diagram indicate sizes in bp. Abbreviations: ma. left, ma. right = left, right arms of the *Mos1 Mariner *transposable element (TE); *AeCPA *promoter = promoter region of the *Ae. aegypti carboxypeptidase A *gene; dcr2 = cDNA fragments corresponding to the *Aa-dcr2 *gene; i = minor intron of the *Ae. aegypti sialokinin I *gene; svA = transcription termination signal derived from the SV40 virus; EGFP = green fluorescent protein marker; 3xP3 = eye tissue-specific promoter. B) Percentage of midgut-specific silencing of *Aa-dcr2 *mRNA among nine different transgenic *Ae. aegypti *lines at 1 day pbm. *Aa-dcr2 *expression levels in midguts of bloodfed females were normalized for gene expression levels of sugarfed females of the lines at the same time point. Bloodmeals were obtained from mice. Each sample consisted of total RNA from a pool of 20 midguts.

We designed a donor plasmid based on the *Mariner Mos1 *transposable element (TE) containing an *Aa-dcr2 *IR expression cassette under control of the bloodmeal inducible, midgut-specific *AeCPA *promoter (Fig. 1A). The donor plasmid was co-injected with a helper plasmid expressing the *Mos1 *transposase [29] into 1780 pre-blastoderm embryos of the *Ae. aegypti *HWE strain. The survival rate was 10.3%. After outcrossing to the HWE recipient strain, 115 G_0 _families were established and their offspring (G_1_) were screened for eye-specific EGFP expression. We selected 10 different mosquito families that produced transgenic offspring, Carb/dcr16, 29, 44, 54, 69, 79, 113, 125, 126, and 146.

### Levels of *Aa-dcr2 *silencing among the transgenic *Ae. aegypti *lines

As an initial molecular characterization we analyzed *Aa-dcr2 *mRNA expression in midguts of nine of the 10 transgenic lines after bloodfeeding by quantitative reverse transcriptase PCR (qRT-PCR). Line Carb/dcr69 was eventually lost during mosquito rearing. One week post-emergence females of the nine lines were bloodfed on mice. Relative *Aa-dcr2 *mRNA accumulation was reduced by >50% in mosquito midguts of lines Carb/dcr16 and Carb/dcr44 at day 1 post-bloodmeal (pbm) as compared to sugarfed control mosquitoes (Fig. 1B). For lines Carb/dcr54, 125, 79, and 29, relative levels of *Aa-dcr2 *mRNA reduction were between 10-45%. On the contrary, for lines Carb/dcr126, 146, and the non-transgenic HWE control relative *Aa-dcr2 *mRNA levels were increased in mosquito midguts. Based on the *Aa-dcr2 *mRNA expression profile of Carb/dcr16 females, we selected this line for further vector competence studies with SINV-TR339EGFP.

### Characterization of the transgene integration site in Carb/dcr16 mosquitoes

**Figure 2:**
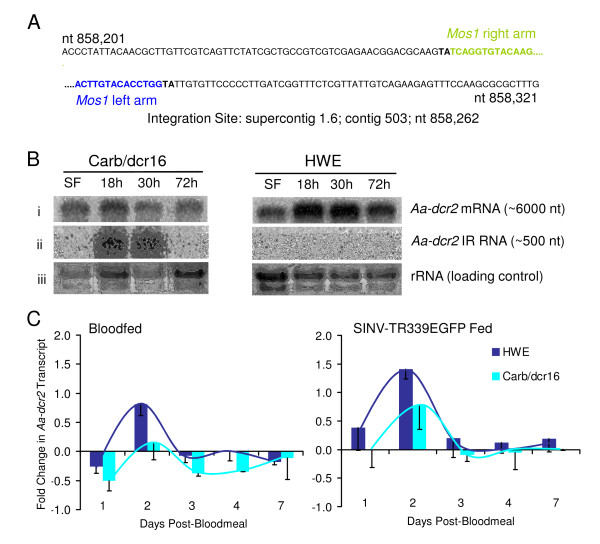
**Molecular characterization of Carb/dcr16 mosquitoes**. A) Genomic DNA sequences flanking the left and right arms of the modified *Mariner Mos1 *TE after its integration into the genome of Carb/dcr16 mosquitoes. In bold: duplicated endogenous *Mos1 *target site; green letters: partial DNA sequence of the right arm of the *Mos1 *TE; blue letters: partial DNA sequence of the left arm of the *Mos1 *TE. B) Northern blot analysis of *Aa*-*dcr2 *mRNA and transgene expression levels in midguts of Carb/dcr16 and HWE control females at 18, 30, and 72 h pbm (SF = midgut RNA of sugarfed females). C) Levels of midgut-specific *Aa-dcr2 *silencing among bloodfed or SINV-TR339EGFP infected Carb/dcr16 and HWE females at 1-7 days pbm. *Aa-dcr2 *expression levels in midguts of bloodfed females were normalized for gene expression levels of sugarfed females at similar time points. Mosquitoes obtained artificial bloodmeals consisting of defibrinated sheep blood. Values below zero indicate silencing of *Aa-dcr2 *and values above zero indicate up-regulation of the gene. Wave-shaped lines represent the *Aa-dcr2 *expression profiles in midguts of Carb/dcr16 and HWE females. Bars represent mean values of three replicates for HWE and two replicates for Carb/dcr16 mosquitoes. Each replicate consisted of total RNA from a pool of 20 midguts (error bars = SEM).

The transgene integration site in the genome of Carb/dcr16 mosquitoes was defined by Genome Walking. We confirmed the stable integration of the *Mos1 *based transgene into the genome of HWE mosquitoes by the fact that DNA sequences flanking the left and right arms of the TE were continuous (Fig. 2A). The TE integration site is in a non-protein encoding region at nucleotide position 858,262 of contig 503, supercontig 1.6. Absence of any other sequences from the Genome Walking libraries strongly suggests that integration of the TE occurred as a single copy.

### Phenotypic analysis of SINV-TR339EGFP

The 720 base-pair coding sequence of the EGFP gene was inserted into a recombinant cDNA clone of SINV-TR339. The marker gene was placed under control of the engineered, duplicated subgenomic promoter that was located upstream of the sequence encoding the viral structural genes. Growth curve analysis of SINV-TR339EGFP in Vero cells revealed an increase in virus titer from 1 × 10^6 ^to 4 × 10^7 ^pfu/ml between 15 and 38 h post-infection (multiplicity of infection: 0.01). Then the titer gradually decreased to 2 × 10^6 ^at 65 h post-infection. The pattern of the growth curve was similar to that observed for the TR339 strain of SINV lacking a duplicated subgenomic promoter [13]. Furthermore, strong EGFP expression was observed among the cells at 38 h post-infection. However, in SINV-TR339EGFP infected tissue such as the mosquito midgut, EGFP expression was often rather low even though virus titers proved to be relatively high (data not shown). This observed discrepancy between viral marker gene expression and actual titers prompted us in the following experiments to base SINV-TR339EGFP detection in mosquitoes on intensity of infection rather than visualization of EGFP expression.

### Evaluation of transgene expression and *Aa-dcr2 *mRNA levels in midguts of Carb/dcr16 females

Detection of a single RNA band corresponding to a size of ~500 nt by Northern blot analysis showed that *Aa*-*dcr2 *derived IR RNA was transcribed in midguts of Carb/dcr16 females 18-30 h after receiving a non-infectious bloodmeal (Fig. 2Bii). A similar signal was not detected at a later time point or in midguts of sugarfed Carb/dcr16 females and in the HWE control. This temporal and spatial expression pattern was in agreement with those observed for other transgenes controlled by the *AeCPA *promoter [23,24]. Hybridization signal intensities for *Aa-dcr2 *mRNA among midgut RNA of bloodfed Carb/dcr16 mosquitoes were considerably weaker at 18-72 h pbm compared to those of bloodfed HWE at similar time points (Fig. 2Bi). This indicates silencing of the RNAi pathway gene in midguts of the bloodfed transgenic mosquitoes. In addition, we assessed the *Aa-dcr2 *mRNA expression profile for Carb/dcr16 mosquitoes during one week by qRT-PCR. *Aa-dcr2 *expression in midguts of bloodfed females followed a wave-like pattern with lowest expression in the transgenic line at days 1, 3 and 4 pbm and maximal expression at day 2 pbm (Fig. 2C). Accumulation of *Aa-dcr2 *mRNA was reduced in midguts of Carb/dcr16 females as compared to the HWE control with the exception of day 7 pbm, a time point when the transgene was no longer expressed. We observed that *Aa-dcr2 *expression profiles were generally less elevated in Carb/dcr16 and HWE mosquitoes that had received an artificial bloodmeal containing defibrinated sheep blood than in mosquitoes that had been allowed to feed on mice (data not shown). After ingestion of a bloodmeal containing SINV-TR339EGFP (titer in the bloodmeal: 2.2 × 10^7 ^pfu/ml), *Aa-dcr2 *mRNA levels in midguts of Carb/dcr16 and HWE followed a similar wave-like pattern. *Aa-dcr2 *mRNA accumulation was substantially increased in Carb/dcr16 and HWE mosquitoes during the one week observation even though the overall level of *Aa-dcr2 *mRNA was still lower in the transgenic females than in HWE. This suggests that replicating SINV-TR339EGFP has triggered the RNAi pathway in the mosquito midgut.

### Effects of *Aa-dcr2 *silencing in the midgut of Carb/dcr16 females on intensity of SINV-TR339EGFP infection, infection rate, and dissemination in an initial experiment

**Figure 3:**
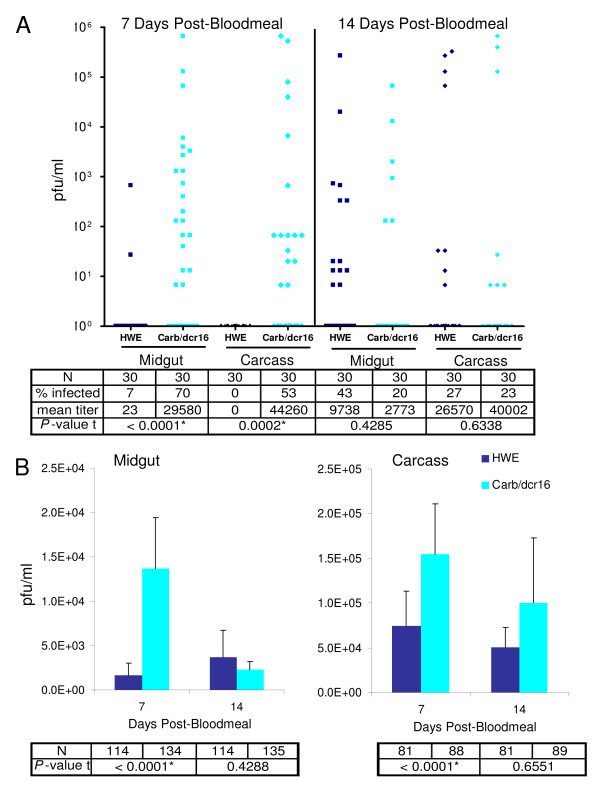
**Intensity of SINV-TR339EGFP infection in Carb/dcr16 and HWE mosquitoes**. A) Raw data of a single experiment in which Carb/dcr16 females were orally challenged with SINV. Each data point represents the virus titer (pfu/ml) in midgut or carcass of an individual mosquito. *P*-values for intensities of virus infection are shown in the table. B) Mean intensities of SINV infection in midguts and carcasses of Carb/dcr 16 and HWE females at 7 and 14 days pbm. Mean values of three experiments are shown. (N = sample size; * = statistically significantly different (α = 0.05); error bars = SEM).

To test whether midgut-specific silencing of *Aa-dcr2 *affects the vector competence for SINV-TR339EGFP, infection intensities and virus infection and dissemination rates were evaluated in Carb/dcr16 mosquitoes. In an initial experiment (virus titer in the bloodmeal: 1.8 × 10^7 ^pfu/ml), midgut infection rate and intensity of virus infection were significantly higher in Carb/dcr16 than in HWE mosquitoes at 7 days pbm (Fig. 3A). We observed that 21/30 Carb/dcr16 females were infected with a ~1300-fold higher mean virus titer than the HWE control. In contrast, only 2/30 HWE mosquitoes had measurable virus infection in their midguts. Accordingly, 53% of the remaining mosquito bodies of Carb/dcr16 females were infected with SINV at 7 days pbm, whereas no HWE carcasses showed any detectable infection. This indicates that midgut infection rate and intensity affect the dissemination potential of the virus to secondary tissues. However, at 14 days pbm the overall SINV infection patterns of Carb/dcr16 females were no longer significantly different from those of the HWE control. These results suggest that SINV-TR339EGFP encountered MIB and MEB in HWE mosquitoes at 7 days pbm, whereas in the RNAi-impaired Carb/dcr16 females these barriers were not evident.

### Effects of *Aa-dcr2 *silencing in the midgut of Carb/dcr16 females on mean intensities of SINV-TR339EGFP infection, infection and dissemination rates

To confirm this observation, we repeated the experiment three more times and assessed mean intensity of SINV infection and midgut infection rates. To reveal mean midgut dissemination rates for the virus, two additional replicates of the experiment were analyzed. SINV-TR339EGFP titers in the bloodmeals ranged from 1.7-2.7 × 10^7 ^pfu/ml. The mean intensity of virus infection in midguts of Carb/dcr16 females (14,000 pfu/ml) was >8-fold higher than in the control at 7 days pbm, which was highly significant (Fig. 3B). Similarly, in the remaining mosquito bodies the difference between HWE and Carb/dcr16 females was statistically significant. In contrast, mean intensities of SINV-TR339EGFP infection did not differ significantly between the transgenic strain and the HWE control at 14 days pbm. At this time point however, virus titers were reduced by 83% in midguts of Carb/dcr16 mosquitoes as compared to seven days earlier. This effect was observed only in the RNAi-impaired Carb/dcr16 mosquitoes. Since SINV titers of carcasses were not increased at 14 days pbm as compared to 7 days pbm, we assume that reduction in the intensity of virus infection in midguts was not caused by virus dissemination to secondary tissues.

**Figure 4:**
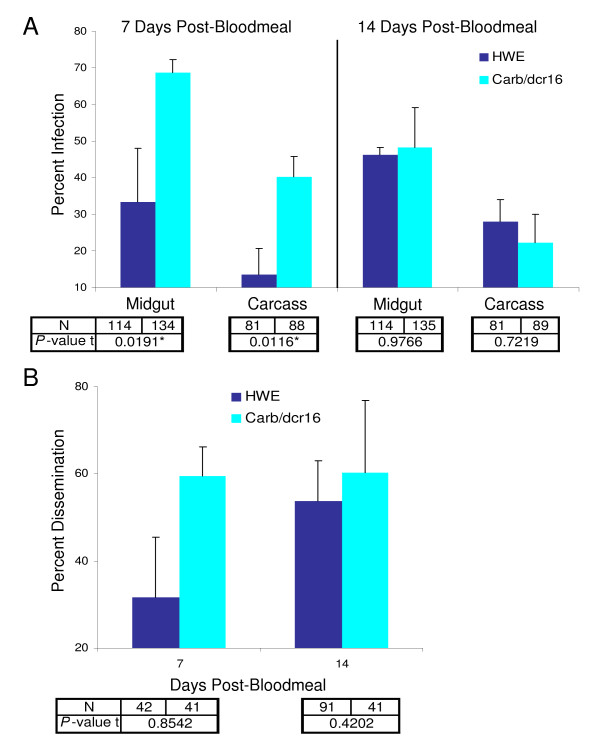
**Infection and dissemination rates of SINV-TR339EGFP in Carb/dcr16 and HWE mosquitoes**. A) Midgut and carcass infection rates of Carb/dcr16 and HWE females with SINV at 7 and 14 days pbm. Mean values of three experiments are shown (N = sample size; * = statistically significantly different; error bars = SEM). B) Dissemination rate of SINV in Carb/dcr16 and HWE females at 7 and 14 days pbm. Mean values of two experiments are shown (N = sample size; error bars = SEM). Infection and dissemination rates were determined by plaque assays.

The mean midgut infection rate with SINV-TR339EGFP was significantly higher among Carb/dcr16 mosquitoes (69%) than among the HWE control (33%) at 7 days pbm (Fig. 4A). As the standard error in Fig. 4A predicts, midgut infection rates of the HWE mosquitoes had a relatively high variability between experiments. Clearly, in the RNAi-impaired Carb/dcr16 females the midgut infection rates did not fluctuate as strongly. This suggests that HWE responded more sensitively to changes in virus dose present in bloodmeals of different challenge experiments. At 7 days pbm the mean infection rate of the carcasses was significantly lower among HWE than among Carb/dcr16 females. At 14 days pbm mean midgut and carcass infection rates no longer differed significantly between both mosquito strains. In Carb/dcr16 females mean infection rates were decreased by 20% at 14 days pbm compared to those at 7 days pbm even though in HWE they were increased by ~20% (Fig. 4A). This is in accordance with the data obtained from the analysis of midgut infection intensity (Fig. 3B), showing that in the transgenic mosquitoes SINV was diminished in midguts after 7 days pbm.

When comparing the mean dissemination rates of SINV-TR339EGFP between HWE and Carb/dcr16, we only considered mosquitoes having infections in both midgut and carcass at 7 or 14 days pbm. In both mosquito strains, virus dissemination rates followed a pattern similar to the midgut infection rates at 7 days pbm (Fig. 4B). Differences were not statistically significant between Carb/dcr16 and HWE mosquitoes even though dissemination rates were about twice as high in Carb/dcr16 females (60%) at 7 days pbm. The lack of statistical significance could be due to the smaller sample sizes available for this experiment. However, our data suggest that dissemination rates for SINV-TR339EGFP are dependent on the virus dose ingested by the mosquito.

### Survival rates of Carb/dcr16 females after infection with SINV-TR339EGFP

**Figure 5:**
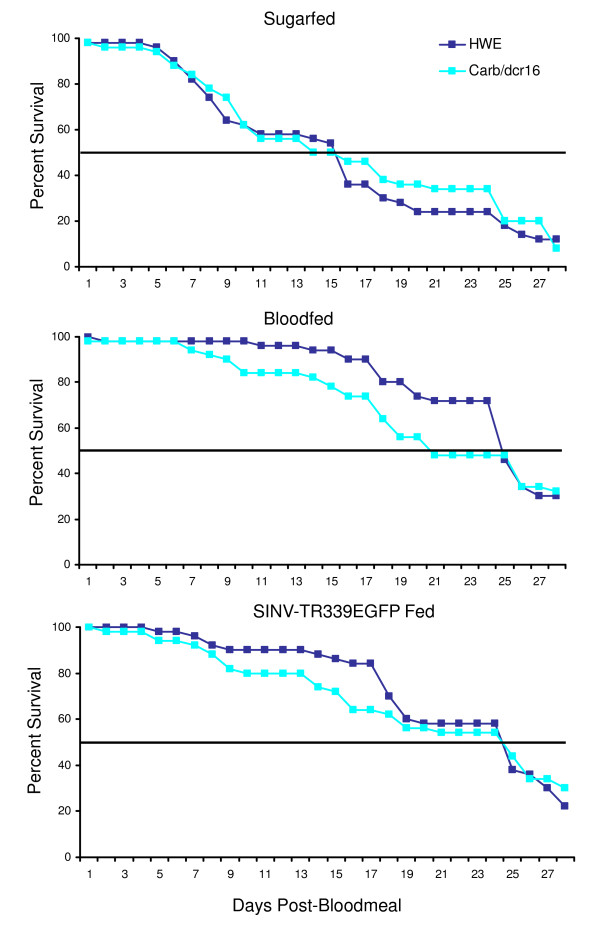
**Survival rates of sugarfed, bloodfed or SINV-TR339EGFP fed Carb/dcr16 and HWE females**. Daily survival rates were monitored for 28 days among one week-old females that had received a non-infectious or SINV-TR339EGFP containing bloodmeal. Sugarfed females were used as control. Bold lines indicate 50% survival.

We evaluated the effect of midgut-specific impairment of the RNAi pathway on the longevity of mosquitoes once they were infected with SINV-TR339EGFP. So far our data have shown that at 7 days pbm the RNAi pathway-impaired mosquitoes contained higher doses of the virus than the HWE control. We monitored the survival rate of mosquitoes for four weeks after bloodfeeding. Bloodfeeding appeared to have a beneficial effect for both Carb/dcr16 and HWE females since 50% of the insects were still alive at day 25 pbm whereas of the sugarfed control only 20% were alive at the same time point (Fig. 5). When both mosquito strains were infected with SINV-TR339EGFP (titer in the bloodmeal: 2.7 × 10^7 ^pfu/ml), their longevity was not affected in comparison to non-infected, bloodfed mosquitoes. The survival curves looked similar for Carb/dcr16 and HWE females, indicating that SINV infection did not cause an obvious fitness cost in the RNAi-impaired mosquitoes.

## Discussion

This study demonstrates for the first time a transgenic approach to impair the endogenous RNAi pathway in midgut tissue of *Ae. aegypti*. Following the principle of activating the RNAi pathway in specific tissues during digestion of a bloodmeal [24,25,30], we generated mosquitoes expressing an *Aa-dcr2 *targeting IR RNA in the midgut to trigger the RNAi pathway against itself. Thus, we developed a novel tool to study arbovirus-mosquito interactions at the molecular level. With current genetic tools it is not possible to generate a stable gene-knockout mutant of *Ae. aegypti *via homologous recombination (A.W.E. Franz, N. Jasinskiene, M.R. Smith, K.E. Olson and A.A. James, unpublished results). In addition, although intrathoracic injection of dsRNA has been shown to be sufficient to manipulate the RNAi pathway in mosquitoes [2,3,6,24,25] the strategy presented here bears several advantages. 1) Injuries caused by intrathoracic injection of dsRNAs are eliminated, preventing non-specific triggering of other immune pathways and/or reduced longevity of the insect. 2) Off-target effects caused by high doses of injected dsRNAs dispersed throughout the mosquito body are avoided. 3) Precise temporal and spatial gene targeting is ensured.

*Aa-dcr2 *acts at the beginning of the initiation phase of the siRNAi pathway by cleaving long dsRNA molecules into ~21 bp duplexes. With the support of *Aa-r2d2 *these siRNA duplexes are inserted into the RISC complex [31]. When silencing *Aa-dcr2 *using an IR RNA with sequence homology, we expected *Aa-dcr2 *mRNA levels in the cell to diminish over time, which would result in depletion of dicer2 protein. Eventually, there would be insufficient dicer2 enzyme available to maintain the RNAi pathway in a functional state. Based on the pattern of *AeCPA *promoter-based expression, impairing of the RNAi pathway was supposed to last for only 36 h during digestion of the bloodmeal in the midgut. Before the onset of *Aa-dcr2 *mRNA silencing in midgut cells of Carb/dcr16 females, most likely there were sufficient quantities of dicer2 protein synthesized, which could turn the RNAi mechanism against itself. Possibly during the entire 36 h period of RNAi silencing certain quantities of functional dicer2 prevailed in the midgut cells so that the pathway was compromised in its efficiency and capacity but never completely shut off. Similar lack of complete inhibition of RNAi was observed before when transiently silencing *dcr2 *in Drosophila S2 cells [27]. This could explain the pattern of the *Aa-dcr2 *mRNA expression profiles in Carb/dcr16 females, where the efficiency of *Aa-dcr2 *mRNA silencing fluctuated over time but its expression was never eliminated. Moreover, infection with SINV resulted in increased *Aa-dcr2 *mRNA accumulation in Carb/dcr16 females, showing that the midgut epithelial cells were still able to mobilize additional dicer2 protein, even though the pathway was impaired in the midgut tissue. Increase in *Aa-dcr2 *mRNA accumulation confirms earlier findings that the TR339 strain of SINV triggers the RNAi pathway in *Ae. aegypti *[3]. However, no mechanism for *Aa-dcr2 *induction has been described so far. We have no clear explanation as to why at 2 days pbm *Aa-dcr2 *mRNA levels were increased in both HWE and Carb/dcr16 females. We observed that levels of transgenic *Aa-dcr2 *silencing varied considerably between the different transgenic mosquito lines that were initially tested. This could be caused by corresponding variations in *Aa-dcr2 *IR RNA expression levels. Based on previous observations with transgenic mosquitoes expressing a marker gene in midgut tissue (A.W.E. Franz, K.E. Olson, A.A. James, unpublished results), the TE integration site in the genome of the mosquito can strongly affect gene-of-interest expression levels.

Even though maximal silencing of *Aa-dcr2 *in midguts of SINV-TR339EGFP infected Carb/dcr16 females appeared to be no more than ~50%, it had profound effects on intensity of infection, midgut infection and dissemination rates of the virus at 7 days pbm. Average virus titers in midguts increased from 1750 pfu/ml in HWE to 14,000 pfu/ml in Carb/dcr16 mosquitoes. Accordingly, midgut infection rates increased from 33% (HWE) to 69% (Carb/dcr16) and virus dissemination rates from 30% (HWE) to 60% (Carb/dcr16). These data suggest that the RNAi pathway in the mosquito midgut tightly controls SINV infection by modulating its replication. Thus, MIB and MEB for SINV-TR339EGFP in *Ae. aegypti *were virus dose-dependent and in this way affected by the RNAi pathway. Whereas a virus dose-dependent MEB has been reported for the TR339 strain of SINV, no MIB has been observed for this virus [9,13]. Despite the fact that the authors used another mosquito strain in their studies, they also used a non-EGFP expressing virus and higher virus concentrations in their bloodmeals, ranging from 10^8^-10^9 ^pfu/ml. In our study the virus concentrations in bloodmeals ranged from 1.7-2.7 × 10^7 ^pfu/ml. In the presence of a functional RNAi mechanism as in HWE mosquitoes, the lower virus concentration in the bloodmeal was probably approaching the threshold for midgut infection. In the RNAi pathway impaired Carb/dcr16 mosquitoes however, this virus concentration was sufficient to cause productive midgut infections.

Between 7 and 14 days pbm a strong reduction of virus infection intensity was observed in midguts of Carb/dcr16 mosquitoes, causing a decrease in average SINV titers from 14,000 to 2400 pfu/ml. Such strong reduction of virus infection intensity was not observed in the RNAi pathway competent HWE control. After 7 days pbm the RNAi pathway in Carb/dcr16 mosquitoes was no longer compromised as it was during virus acquisition. It appears that the RNAi mechanism, when functional, down-regulated the unusually high SINV concentration in midguts of the transgenic mosquitoes to levels similar to those of the HWE control. This strongly suggests that the task of the RNAi pathway in the mosquito midgut is to keep arbovirus replication at a level that can be tolerated by the mosquito. Modulation of arbovirus infections in mosquitoes has been reported for several virus-vector combinations and research of the last few years eventually confirmed that the RNAi pathway of the mosquito is a major driving force behind this modulation [2,3,6,14,16,32]. Nevertheless, recent studies indicate that other innate immune pathways, such as JAK-STAT and/or Toll also contribute to the modulation of arbovirus infections in insects [33-37].

Since a proposed role for the RNAi pathway in mosquitoes is to protect the insect from pathogenic effects of replicating arboviruses [4-6], we investigated whether SINV-TR339EGFP causes such effects in HWE or Carb/dcr16 mosquitoes. Our survival curve data indicate that the initial increase in virus titer in Carb/dcr16 females did not cause obvious pathogenic effects. It needs to be pointed out that after 7 days pbm the RNAi pathway was no longer impaired in midguts of Carb/dcr16 mosquitoes and the intensity of infection was strongly modulated. Thus, the RNAi pathway activation in the transgenic mosquito line could have been similar to that in the control for the latter 21 days of the survival study. Our observations confirm those by Campbell and co-workers [3] that transient silencing of the RNAi pathway in *Ae. aegypti *did not affect longevity of the mosquitoes for seven days after infection with SINV. However, several authors have described pathological effects caused by alphaviruses in mosquito midguts and salivary glands, claiming that these effects could be virus dose-dependent [38-41]. Moreover, systemic expression of a potent RNAi pathway suppressor via a recombinant SINV severely reduced the survival rate of mosquitoes [4,5]. Thus, it might be necessary to knockout the RNAi pathway in the insect to reveal long-term effects of a compromised, antiviral immune pathway on mosquito fitness.

## Conclusions

We generated transgenic mosquitoes that have an impaired RNAi pathway in the midgut following ingestion of a bloodmeal. These mosquitoes, Carb/dcr16, represent a novel tool to study arbovirus-mosquito interactions at the molecular level. Temporal impairment of the RNAi pathway in the midgut epithelium of Carb/dcr16 mosquitoes significantly increased the infection intensity of SINV-TR339EGFP, thereby allowing the virus to overcome MIB and MEB. Thus, both barriers, which are affected by the endogenous RNAi mechanism, appear to be virus dose-dependent phenomena for this SINV strain in *Ae. aegypti*. Furthermore, the infection pattern of SINV in Carb/dcr16 females suggests that the RNAi pathway is modulating virus replication in the midgut to prevent the virus from reaching harmful concentrations in the insect. As a consequence, longevity of SINV-TR339EGFP infected mosquitoes was similar to that of non-infected ones. Overall, our data confirm that the mosquito midgut is the central organ that determines vector competence for arboviruses.

## Future Directions

Using Carb/dcr16 mosquitoes, we plan to evaluate effects of RNAi pathway impairment in the midgut on infection patterns of dengue and Chikungunya viruses, which are naturally transmitted by this mosquito species.

## Methods

### Transgene design and generation of transgenic *Ae. aegypti*

Five hundred base-pair cDNA fragments corresponding to the ribonuclease I domain encoding region of *Aa-dcr2 *were inserted in sense and anti-sense orientations into *p*SLfa1180fa. Both fragments were separated by the small intron of the *Aa-sialonkinin I *gene [42]. The resulting inverted-repeat (IR) DNA was placed downstream of the *AeCPA *promoter and a SV40 transcription termination signal was added at the 3' terminus of the IR construct. This construct was then inserted into the non-autonomous *Mariner Mos1 *TE containing an eye tissue-specific EGFP expression cassette to allow easy identification of individual mosquitoes harboring the TE [43]. Transgenic mosquitoes were generated as described earlier [24,44,45] using the Higgs White Eye (HWE) strain of *Ae*. *aegypti *as recipient [46]. Mosquitoes received bloodmeals from mice following Colorado State University Institutional Animal Care and Use Committee (IACUC) regulations (IACUC protocol: 09-1365A-01). Mosquitoes were reared in a BSL2 insectary at 28°C and 80% relative humidity. Hemizygous Carb/dcr16 mosquitoes were maintained as an inbred colony. In the experiments intercrossed generations G_5 _to G_8 _were used among which 60-80% of the individuals were transgenic based on fluorescent eye marker expression.

### Characterization of the transgene integration site

The transgene integration site in Carb/dcr16 mosquitoes was characterized by Genome Walking using the GenomeWalker Universal Kit and Advantage2 Polymerase (Clontech, Palo Alto, CA) as described before [25]. Gene-specific primers for the detection of genomic DNA surrounding the *Mariner Mos1 *left arm in Carb/dcr16 mosquitoes were maLeft FWD (5'caattatgacgctcaattcgcgccaaac3') and maLeft_nested FWD (5'gtggttcgacagtcaaggttgacacttc3'). To detect genomic DNA surrounding the right arm of the TE primers maRight FWD (5'gcagtttccaatcgcttgcgagagatg3') and maRight_nested FWD (5' atgagttgaacgagaggcagatggagag3') were used.

### Detection of transgene expression levels by Northern blot analysis

Expression of the IR RNA targeting *Aa-dcr2 *in Carb/dcr16 mosquitoes was evaluated by Northern blot analysis. Using TRIzol Reagent (Invitrogen, Carlsbad, CA) total RNA was extracted from pools of 120 midguts of transgenic and HWE control females that had received a sugarmeal or bloodmeal 18, 30 or 72 h before. For each sample 5 μg of RNA was separated electrophoretically in a 1.2% agarose gel and blotted onto a positively charged nylon membrane (Applied Biosystems, Foster City, CA). The blot was hybridized with a random primed 500 bp ^32^P-dCTP labeled cDNA probe (3000 ci/mmol), which was prepared using the DECAprime II DNA Labeling Kit (Applied Biosystems). The sequence of the probe corresponded to the *Aa-dcr2 *IR effector of Carb/dcr16 mosquitoes.

### Quantification of Aa-*dcr2 *mRNA levels

Quantitative reverse transcriptase PCR (qRT-PCR) was conducted to determine *Aa*-*dcr2 *mRNA levels in midguts of females. Midguts from 20 females were dissected at 1, 2, 3, 4, and 7 days pbm and stored in TRIzol Reagent (Invitrogen) at -80°C until total RNA was extracted according to the manufacturer's protocol. qRT-PCR was performed using the QuantiFast SYBR Green RT-PCR kit (Qiagen, Valencia, CA) and the iQ5 Real-Time PCR Detection System (BioRad, Herciles, CA). To quantify *Aa*-*dcr2 *cDNAs, primers dcr2 qFWD (5'tcggaaatttcaacgatagctcgtaaca3') and dcr2 qREV (aattcgcgtaggaaccgtactccggatt3') were used. The RT reaction was conducted for 10 min at 50°C followed by a PCR reaction (5 min at 95°C and 35 cycles of 10 s at 95°C and 30 s at 60°C). *Aa-dcr2 *standards consisted of serially diluted cDNA clones containing the *Aa-dcr2 *PCR product (181 bp in size) and were used to derive the copy number per ng of total RNA. Resulting *Aa-dcr2 *copy numbers obtained from midgut RNA of bloodfed or virus-infected females were normalized for copy numbers obtained from midgut RNA of sugarfed females.

### Oral infection of Carb/dcr16 and HWE mosquitoes with SINV-TR339EGFP

Prior to a bloodfeeding experiment mosquitoes were reared on raisins and water. A large 2.5 L carton typically contained 125 females and 10 males. Raisins and water were removed from the cartons 36 h and 5 h, respectively before bloodfeeding. To infect females with SINV-TR339EGFP one week post-emergence, defibrinated sheep blood was mixed at a 1:1 ratio with virus freshly harvested from Vero cell culture medium. SINV titers in bloodmeals ranged from 1.7 to 2.7 × 10^7 ^pfu/ml. HWE and Carb/dcr 16 females were fed for 1 h using one glass feeder per carton, which contained 2 ml of bloodmeal maintained at 37°C. After bloodfeeding, the mosquitoes were sorted for females that were three quarters or fully engorged. These individuals were further reared in 470 ml cartons (40 females/carton) and fed with sucrose and water until further analysis.

### Propagation of SINV-TR339EGFP and determination of virus titers by plaque assay

SINV-TR339EGFP virus stocks were generated from an infectious cDNA clone that contained the EGFP marker gene under control of a duplicated sub-genomic promoter located upstream of the coding sequence for the structural genes [3]. Virus titers from individual midguts and bodies were determined by plaque assay at 7 and 14 days pbm as described before [2]. Briefly, samples were homogenized in 0.5 ml MEM medium with 7% FBS and filtered with Acrodisc HT Tuffryn 0.2 μm syringe filters (Pall Life Sciences, East Hills, NY). Vero cells were seeded into 24-well plates and left for three days to achieve confluence. Cells were infected with 10-fold serial dilutions of individual midgut or carcass homogenates. Cells were incubated for 1 h at 37°C before overlaid with an agarose-nutrient mixture [1× Medium 199 (Sigma-Aldrich, St. Louis, MO), 10% FBS, 4% NaHCO_3_, 0.5% MEM vitamins, 0.5% MEM amino acids (Mediatech Inc., Manassas, VA)]. The plates were incubated at 37°C for 4 days. Cells were then stained with MTT (3- [4,5-dimethylthiazol-2-yl]-2,5-diphenyltetrazolium bromide) (Sigma-Aldrich, St. Louis, MO), incubated at 37°C for 24 h and the number of plaques was counted for each sample. Virus titers of individual mosquitoes were calculated as pfu/ml.

### Survival curve of *Ae. aegypti*

Seven day-old Carb/dcr16 and HWE females were either fed with a non-infectious bloodmeal or with a bloodmeal containing SINV-TR339EGFP. After bloodfeeding, 50 mosquitoes of each treatment were put into 470 ml cardboard containers and provided with sugar and water. A control consisting of females that were sugarfed only was included in the experiment. For a period of 28 days after bloodfeeding the daily number of surviving mosquitoes in each container was recorded.

### Statistical analysis

Statistical analyses were performed using SAS Statistical Analysis Software (SAS Institute Inc., Cary, NC). The MIXED procedure was used for restricted maximum likelihood parameter estimation with incomplete data. Aa-*dcr2 *ratios and SINV-TR339EGFP infection levels were normalized using a log10 transformation. Aa-*dcr2 *ratios, virus infection levels, and virus infection/dissemination rates were then analyzed using the least-squares means test followed by pair-wise comparisons with the Tukey-Kramer test.

## Authors' contributions

CCHK designed and performed the qRT-PCR assays, virus challenge and survival experiments, analyzed the data and wrote the manuscript. JP assisted with sample preparations, qRT-PCR assays, mosquito rearing and virus challenge experiments. ISV performed the Northern blot. KEO conceived the study, analyzed the data and edited the manuscript. AWEF conceived the study, generated the IR effector construct and the transgenic mosquitoes, performed the Genome Walking experiment, analyzed the data and edited the manuscript. All authors read and approved the final manuscript.
